# Muscle cells and motoneurons differentially remove mutant SOD1 causing familial amyotrophic lateral sclerosis

**DOI:** 10.1111/j.1471-4159.2011.07298.x

**Published:** 2011-07

**Authors:** Elisa Onesto, Paola Rusmini, Valeria Crippa, Nicola Ferri, Arianna Zito, Mariarita Galbiati, Angelo Poletti

**Affiliations:** * Dipartimento di Endocrinologia, Fisiopatologia e Biologia Applicata, and Centre of Excellence on Neurodegenerative Diseases, Università degli Studi di MilanoMilano, Italy; † InterUniversity Center on Neurodegenerative Diseases of the Universities of FlorenceRome and Milan, Italy; ‡ Dipartimento di Scienze Farmacologiche, Università degli Studi di MilanoMilano, Italy

**Keywords:** amyotrophic lateral sclerosis, autophagy, motoneuron diseases, muscle cells, proteasome, SOD1

## Abstract

Amyotrophic lateral sclerosis (ALS) is a fatal motoneuronal disease which occurs in sporadic or familial forms, clinically indistinguishable. About 15% of familial ALS cases are linked to mutations of the superoxide dismutase 1 (SOD1) gene that may induce misfolding in the coded protein, exerting neurotoxicity to motoneurons. However, other cell types might be target of SOD1 toxicity, because muscle-restricted expression of mutant SOD1 correlates with muscle atrophy and motoneurons death. We analysed the molecular behaviour of mutant SOD1 in motoneuronal NSC34 and muscle C2C12 cells. We found that misfolded mutant SOD1 clearance is much more efficient in muscle C2C12 than in motoneuronal NSC34 cells. Mutant SOD1 forms aggregates and impairs the proteasome only in motoneuronal NSC34 cells. Interestingly, NSC34 cells expressing mutant SOD1 are more sensitive to a superoxide-induced oxidative stress. Moreover, in muscle C2C12 cells mutant SOD1 remains soluble even when proteasome is inhibited with MG132. The higher mutant SOD1 clearance in muscle cells correlates with a more efficient proteasome activity, combined with a robust autophagy activation. Therefore, muscle cells seem to better manage misfolded SOD1 species, not because of an intrinsic property of the mutant protein, but in function of the cell environment, indicating also that the SOD1 toxicity at muscle level may not directly depend on its aggregation rate.

Amyotrophic lateral sclerosis (ALS) is a fatal adult onset neurodegenerative disease. ALS is characterized by the progressive loss of motoneurons in the cerebral cortex, in the brain stem and in the anterior horns of the spinal cord (Pasinelli and Brown 2006). ALS has been reported to occur in sporadic and familial (fALS) forms, which are clinically indistinguishable. Up to now, several genes have been linked to fALSs. One of these is the gene coding for the superoxide dismutase 1 (SOD1), which accounts for approximately 15% of fALS cases. Most SOD1 alterations are point mutations, which are thought to destabilize protein conformation leading to misfolding (Seetharaman *et al.* 2009). The misfolded SOD1 may then perturb several motoneuronal functions. It has been proposed that misfolded proteins may either acquire aberrant enzymatic activities, impair axonal transport, form intracellular aggregates, saturate the proteasome, induce mitochondrial dysfunctions, etc. (Pasinelli and Brown 2006; Cozzolino *et al.* 2008; Seetharaman *et al.* 2009). Several studies, with conflicting results, have been performed to understand whether this toxicity is confined to motoneurons, or other cell types (such as glial cells, microglia, Schwann cells, and/or muscle cells) may be affected, thus playing a role in the disease. With regard to the muscle cells, skeletal muscle dysfunctions and neuromuscular junction degeneration occur long before disease onset and motoneuron death (Frey *et al.* 2000; Fischer *et al.* 2004). Moreover, the expression of mutant SOD1 in skeletal muscle induces progressive muscle atrophy (Dobrowolny *et al.* 2008b), and the damaged muscle cells also have impact on the survival of motoneurons located in the anterior horns of the spinal cord (Wong and Martin 2010). This suggests that motoneuronal degeneration might occur as a consequence of the loss of the target muscle cells, and the consequent degeneration of the neuromuscular junction and of the axonal processes. A contribution of the lack of muscle-derived growth factors, which normally preserve motoneurons from death, cannot also be excluded (Musaro *et al.* 2001; Kaspar *et al.* 2003; Dobrowolny *et al.* 2005, 2008a; Palazzolo *et al.* 2009). However, motoneuronal restricted expression of mutant SOD1 is also sufficient to cause ALS (Wang *et al.* 2008). Therefore, both motoneurons and muscle may be target of mutant SOD1 toxicity and possibly both may synergistically contribute to accelerate the onset and the progression of ALS.

We already characterized the molecular behaviour of mutant SOD1 in immortalized motoneurons and described potential mechanisms by which this misfolded protein may become neurotoxic (Sau *et al.* 2007; Crippa *et al.* 2010b). We found that the formation of high molecular weight species of mutant SOD1 generates intracellular aggregates, which correlates with a marked proteasome impairment and alters SOD1 intracellular distribution (Sau *et al.* 2007). This also decreases SOD1 bioavailability resulting in a deprotection from free radical species and the consequent damage to genomic DNA (Sau *et al.* 2007). It is unknown whether the toxicity of mutant SOD1 in muscle cells is caused by the same molecular toxic mechanisms active in motoneurons. Moreover, it is unclear whether muscle cells may be able to better manage the misfolded SOD1 species, thought to be formed as an intrinsic property of the mutant protein, and occurring independently from the cell environment (Dobson 2003).

To this purpose, we compared the biochemical behaviour and degradative processes of wt and mutant SOD1 protein in two different cell models: an immortalized motoneuronal cell line (NSC34) and a muscle cell line (C2C12) maintained either in proliferating or differentiating conditions. The results have shown that misfolded mutant SOD1 is cleared at much faster rate in muscle cells than in motoneuronal cells, preventing the intracellular accumulation of insoluble SOD1 species in muscle cells. The increased clearance of mutant SOD1 in muscle cells is apparently caused by a more efficient proteasome activity, combined with a robust activation of the autophagic system.

## Material and methods

### Materials

All chemicals have been obtained from Sigma (Sigma Aldrich, Milano, Italy). Restriction enzymes and ligase have been obtained from GE Healthcare (Little Chalfont, Buckinghamshire, UK).

### Plasmids

pEGFP-N1 encodes the GFPmut1 variant, a red-shifted variant, of soluble wild-type green fluorescent protein (GFP) (Clontech Laboratories Inc., Mountain View, CA, USA). pDsRed-Monomer-C1 (Clontech Laboratories Inc.) encodes DsRed-Monomer, a monomeric mutant derived from the tetrameric *Discosoma sp.* red fluorescent protein DsRed (λ_ex_ = 557 nm, λ_em_ = 592 nm). pCDNA3-wtSOD1 and pCDNA3-G93A-SOD1 express wtSOD1 and mutant G93A-SOD1 (Tortarolo *et al.* 2004). pECFP-wtSOD1 and pECFP-G93A-SOD1 express wt and mutant human SOD1 tagged with cyan fluorescent protein (CFP) (Sau *et al.* 2007). GFPu (from R. Kopito, USA) and YFPu express a CL1 tagged-GFP or tagged-YFP respectively, two proteasome activity reporter proteins (Rusmini *et al.* 2007; Sau *et al.* 2007). The plasmids pEGFP-wtSOD1 and pEGFP-G93A-SOD1 express GFP-tagged human SOD1 (Crippa *et al.* 2010b). pRFP-LC3 expresses a RFP-tagged rat LC3 (from A. Tolkovsky, Great Britain) (Bampton *et al.* 2005). pDest-mCherry-p62 expresses a Cherry-tagged p62 (from Terje Johansen, University of TromsØ, Norway).

### Cell cultures and transfection

The immortalized motoneuronal NSC34 cell line (Cashman *et al.* 1992) has been routinely maintained and transfected with Lipofectamine (Invitrogen, San Giuliano Milanese, Italy)/transferrin (Sigma Aldrich; 2 : 1) as previously described (Simeoni *et al.* 2000).

The myoblast C2C12 cell line was originally obtained from American Type Culture Collection (Rockville, MD, USA). The cell line was routinely maintained in Dulbecco's modified Eagle's medium (Biochrom KG, Berlin, Germany) supplemented with 1 mM glutamine, 1 mM sodium pyruvate, 100 U/mL penicillin, 100 μg/mL streptomycin, and 10% fetal bovine serum (Invitrogen) at 37°C with 5% CO_2_. Differentiation in muscle C2C12 (C2C12-D) cultures was induced by replacing the growth medium with differentiation medium (2% horse serum, Invitrogen, in Dulbecco's modified Eagle's medium) after the cells reached 70% confluence. Muscle C2C12 cells have been routinely transfected with Lipofectamine 2000 (Invitrogen) following Manufacturers’ instructions.

### Fluorescence and microscopy

Motoneuronal NSC34 cells was plated at 70 000 cells/mL, muscle C2C12 cells at 60 000 cells/mL, in 12-well multiwell plates containing 18-mm glass coverslips; after 24 h the cells have been transfected with 1 μg of plasmids coding for GFP-wtSOD1, GFP-G93A-SOD1, CFP-wtSOD1 or CFP-G93A-SOD1. Co-transfections were performed adding 0.05 μg of plasmid coding for YFPu or 0.3 μg of plasmid coding for mRFP-LC3 or mCherry-p62. For motoneuronal NSC34 cells were added 3 μL of transferrin solution and 2 μL of Lipofectamine; for muscle C2C12 cells 4 μL of Lipofectamine 2000 were added. Cells were allowed to grow for 48 h and then fixed first in phosphate buffer with 4% paraformaldehyde and 4% sucrose, and then in cold methanol. Nuclear staining was achieved by incubation with 4′,6-diamino-2-phenylindole (DAPI) [0.25 μg/mL in phosphate-buffered saline (PBS)] for 1 min at 20°C. The coverslips were mounted in Mowiol® (Calbiochem, San Diego, CA, USA) and examined with an Axiovert 200 microscope (Zeiss Instr., Oberkochen, Germany) equipped with fluorescein isothiocyanate (FITC)/tetramethylrhodamine isothiocyanate (TRITC)/DAPI or with CFP/YFP sets of filters. Fluorescence images were captured with a Photometric CoolSnap CCD camera (Ropper Scientific, Trenton, NJ, USA). Images were processed using Metamorph software (Universal Imaging, Downingtown, PA, USA).

To evaluate the presence of SOD1 aggregates, GFP-SOD1 (wt or G93A) expressing cells were analysed by fluorescence microscopy 48 h after transfection. GFP-SOD1 aggregates were estimated using a PL 10×/20 eyepiece with graticules (100 mm × 100 mm in a 100 grid divisions). Transfected cells were evaluated by their staining pattern as diffusely labelled, or as containing aggregates. The percentage of GFP-SOD1 cells with aggregates was obtained dividing the number of cells bearing GFP-SOD1 aggregates by the total number of transfected cells. At least 60 cells/ field were counted, and three field for each coverslips were analysed. The experiments were performed in triplicate.

### Western blot analysis and filter retardation assay

For western blot and filter retardation assays, cells were plated in 12-well multiwells at 80 000 cells/mL density for motoneuronal NSC34 cells, and at 65 000 cells/mL density for muscle C2C12 cells. Transient transfections were performed co-transfecting 1 μg of pCDNA3, wt or G93A-SOD1 plasmids with 0.05 μg of YFPu plasmids for each sample. Motoneuronal NSC34 cells were transfected with 3 μL of transferrin solution and 2 μL of Lipofectamine for each sample, whereas for muscle C2C12 cells were added 4 μL of Lipofectamine 2000. To inhibit proteasome activity, MG132 (Sigma Aldrich) was used at doses of 10 μM for 15 h. After 48 h from transfection, cells were harvested and centrifuged 5 min at 100 *g* at 4°C; the pellets of cells were resuspended in PBS (added of a protease inhibitors cocktail, Sigma Aldrich) and homogenized using slight sonication. Total proteins were determined with the bicinchoninic acid method (BCA assay, Pierce, Rockford, IL, USA). Western immunoblot analysis was performed on 12% sodium dodecyl sulfate–polyacrylamide gel electrophoresis loading 30 μg of total proteins. Samples were then electro-transferred to nitrocellulose membranes (Trans-blot, Bio-Rad Laboratories, Hercules, CA, USA) using a liquid transfer apparatus (Bio-Rad Laboratories). Nitrocellulose membranes were treated with a blocking solution containing 5% non-fat dry milk in Tween-TBS (TBS-T, 20 mM TrisHCl, pH 7.5, 0.5 M NaCl, 0.05% Tween-20) for 1 h and then incubated with the primary antibodies: (a) peroxidase labelled goat anti-GFP (Vector Laboratories, Burlingame, CA, USA; dilution 1 : 5000 in TBS-T) to detect YFPu; (b) rabbit polyclonal anti-LC3 (Sigma Aldrich; dilution 1 : 1000 in milk); (c) rabbit polyclonal anti-Cu/Zn superoxide dismutase SOD1 (SOD-100; Assay Designs; dilution 1 : 1000 in milk) to detect the wt and G93A-SOD1 proteins; (d) goat polyclonal anti-Actin (Actin I-19; Santa Cruz Biotechnology, Santa Cruz CA, USA, dilution 1 : 1000 in milk) to detect total actin. Immunoreactivity was detected using the following secondary peroxidase-conjugated antibodies: goat anti-rabbit (sc-2004; Santa Cruz Biotechnology) was used to identify the anti-SOD1 and the anti-LC3; donkey anti-goat (sc-2020; Santa Cruz Biotechnology) was used to identify the anti-Actin antibody. The immunoreactive regions were then visualized using the enhanced chemiluminescence detection kit reagents (ECL; GE Healthcare). The same membranes were subsequently processed with different antibodies to detect the levels of proteins in the same samples loaded on the gel, after stripping for 30 min at 37°C in restore western blotting stripping buffer (Pierce).

Filter retardation assay was performed by sample filtration through a 0.2 μm cellulose acetate membrane (Whatman, Dassel, Germany) using a slot-blot apparatus (Bio-Rad Laboratories) and loading 0.75 μg of total proteins. Slot-blots were probed as described for western blots. Optical intensity of samples assayed with filter retardation assay was detected and analysed using NIH ImageJ software.

### Cytofluorimetric analysis

Samples for cytofluorimetric analysis were obtained from NSC34, C2C12 and C2C12-D cells, plated in 12-well multiwell plates at 90 000 cells/mL density (NSC34 cells) or 60 000 cells/mL density (C2C12 and C2C12-D cells), transfected with (a) 1 μg of pEGFP-N1 plasmid; (b) 1 μg of GFP-SOD1, wt or G93A, plasmid; (c) or co-transfected with 0.1 μg of GFPu and 0.2 μg of DsRed-Monomer and 1 μg of pcDNA3. The transfected cells were harvested and centrifuged 5 min at 100 *g* at 4°C; the cell pellets were resuspended in 400 μL of 4% paraformaldehyde, incubated at 20°C for 10 min on a rotator and then centrifuged 5 min at 100 *g* at 4°C. Once supernatant was aspirated from cell preparation, the pellets was resuspended in 300 μL of PBS. Cell fluorescence was detected using FACS Calibur (BD Pharmingen). Flow cytometry results were analysed using CellQuest (BD Pharmigen) program analysis software.

To detect superoxide production, cells were labelled with MitoSOX™ Red mitochondrial superoxide indicator (Molecular Probes, Eugene, OR, USA). MitoSOX™ reagent stock solution (3 mM) was diluted 1 : 1000 in HBSS/Ca/Mg buffer (10 mM HEPES pH 7.4, 150 mM NaCl, 5 mM KCl, 1 mM MgCl_2_ 1.8 mM CaCl_2_) at 37°C. MitoSOX™ reagent working solution (3 μM) was used to replace cell medium, fully covering the cells and incubating for 1 h at 37°C, keeping the samples protected from light. The medium was removed and samples washed with HBSS at 37°C and then collected in 300 μL of PBS. Cells were analysed in cytofluorimeter analysis without fixing in paraformaldehyde (λ_ex_ = 510 nm, λ_em_ = 580 nm; FL2 channel) (Batandier *et al.* 2002).

### Proteasome activity

Proteasome assays were performed as described by Allen *et al.* (2003). Cells were plated in 6-well multiwells at 80 000 cells/mL density for motoneuronal NSC34 cells, and at 70 000 cells/mL density for muscle C2C12 cells; C2C12-D cells were allowed to differentiate for 48 h. Cells were washed with ice-cold PBS and then harvested and centrifuged at 100 *g* for 5 min, at 4°C. Pellets resuspended in 0.3 mL of proteasome extract buffer (20 mM Tris/HCl, pH 7.4, containing 0.1 mM EDTA, 1 mM 2-mercaptoethanol, 5 mM ATP, 20% v/v glycerol, and 0.04% v/v Nonidet P-40) were homogenized by several passages through a 21-gauge needle, and then centrifuged at 12 000 *g* for 15 min at 4°C, saving the supernatant. Total proteins were determined with the bicinchoninic acid method (BCA assay; Pierce). Proteasome assay reaction mixtures consisted of 50 mM HEPES/KOH, pH 8.0, containing 5 mM EGTA, 100 μg of cells extract protein per mL of assay reaction. The reactions were initiated by adding the appropriate proteasome substrates conjugated with aminomethylcoumarin (AMC) and incubating the reactions at 37°C for 45 min. Proteasome substrates used are: (i) the peptide Suc-LLVY-AMC at 50 μM, to detect chimotryptic activity; (ii) the peptide Z-ARR-AMC at 100 μM, to detect tryptic activity and (iii) the peptide Z-LLE-AMC at 100 μM, to detect post-acidic activity. Hydrolysis of the peptides induces the release of AMC and the resulting fluorescence was measured at 340 nm excitation and 460 nm emission using a spectrofluorimeter (Victor, Perkin Elmer, MA, USA).

### mRNA expression analysis

For real-time PCR analysis cells were plated in 6-well multiwells plates at 80 000 cells/mL density for motoneuronal NSC34 cells and at 65 000 cells/mL density for muscle C2C12 cells. After 24 h, cells have been transfected with 2 μg of plasmids coding for pCDNA3, wt or G93A-SOD1. For motoneuronal NSC34 cells were added 6 μL of transferrin solution and 4 μL of Lipofectamine were added for each sample; for muscle C2C12 cells were added 8 μL of Lipofectamine 2000 for each sample. Forty-eight hours from transfection, cells were harvested in 4 M guanidium isothiocyanate (containing 25 mM sodium citrate pH 7.5, 0.5% sarcosyl and 0.1% 2-mercaptoethanol) and total RNA was isolated by phenol–chloroform extraction, according to Chomczynski and Sacchi (1987). Quantification was carried out by absorption at 260 nm. For reverse transcription, an aliquot of total RNA (1 μg) was treated for 15 min at 20°C with 1 U of DNaseI (Sigma). After having heat-inactivated DNaseI, 1 μg of each sample was reverse-transcribed using the High-Capacity cDNA Reverse Transcription Kit (PE Applied Biosystems, Monza, Italy) according to the manufacturer's instructions, in a 25 μL volume. Primers for selected genes were designed via the primer Express software (PE Applied Biosystems) and purchased from Eurofins MWG Operon (Ebersberg, Germany). The sequences of primers were as follows: mMAP-LC3b: 5′-CGT CCT GGA CAA GAC CA-3′ (forward), 5′-CCA TTC ACC AGG AGG AA-3′ (reverse); mp62: 5′-AGG GAA CAC AGC AAG CT (forward), 3′-GCC AAA GTG TCC ATG TTT CA (reverse); *GAPDH*: 5′-CCA GAA CAT CAT CCC TGC AT-3′ (forward), 5′-CAG TGA GCT TCC CGT TCA-3′ (reverse). The evaluated efficiency of each set of primers was close to 100% for both target and reference gene. Real-time PCR was performed using the ABI Prism 7000 sequence detection system (PE Applied Biosystems) in a 25 μL total volume, using the iTaq SYBR Green Supermix (Bio-Rad Laboratories), using 500 nmol primers. PCR cycling conditions were as follows: 94°C for 10 min, 35 cycles at 94°C for 15 s, and 60°C for 1 min. Melting curve analysis was always performed at the end of each PCR assay to control for specificity. Data were expressed as *C*_*t*_ values and used for the relative quantification of targets with the ΔΔ*C*_*t*_ calculation. To exclude potential bias because of averaging data transformed through the equation 2^−ΔΔ*Ct*^ to give N-fold changes in gene expression, all statistics were performed with Δ*C*_*t*_ values. Each experiment was carried out twice, with four independent samples. Each sample was run in duplicate wells.

### Statistical analysis

Statistical analysis has been performed using one-tailed Student's *t*-test for two group comparisons; one-way or two-way anova for three or more group comparisons using the PRISM software (GraphPad, San Diego, CA, USA).

## Results

As recent data have suggested that the skeletal muscle could be a primary target of mutant SOD1 toxicity in fALS, in this study we analysed the biochemical behaviour of mutant SOD1 and its potential deleterious effects in muscle cells, compared with immortalized motoneurons.

### Biochemical behaviour of mutant SOD1 in motoneuron and muscle cells

**Fig. 1:**
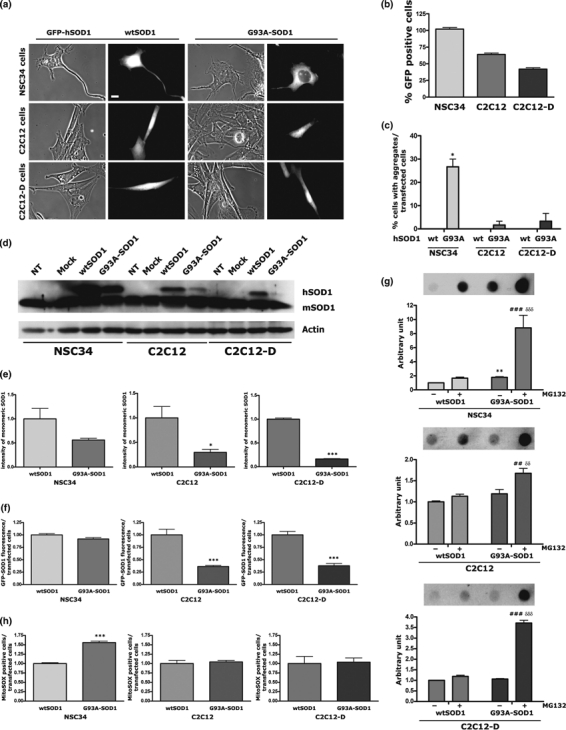
Biochemical behaviour of wt and mutant SOD1 in motoneuronal NSC34 cells and in muscle C2C12 cells. (a) High resolution fluorescent microscopy analysis on cells transfected with GFP-wtSOD1 or GFP-G93A-SOD1. Upper insets, NSC34 cells; middle insets, proliferating C2C12 cells (C2C12 cells); lower insets, differentiating C2C12 cells (C2C12-D cells). GFP λ_ex_ = 488 nm, λ_em_ = 509 nm. Images taken at 63×. Scale bar = 10 μm. (b) Flow cytofluorimetric analysis of NSC34, C2C12 and C2C12-D cells expressing soluble EGFP protein, evaluating the number of EGFP-positive cells in a total of 10 000 cells. (c) Quantification of SOD1 aggregates in NSC34 and C2C12 cells, in proliferating or differentiating status. *t*-Test analysis on wtSOD1 versus G93A-SOD1 transfected cells. *p < 0.05 versus wtSOD1 transfected NSC34 cells. (d) Western blot assay performed on cells lysates of NSC34 or C2C12 cells. NT, untransfected cells; mock, pcDNA3 transfected cells; wtSOD1, human wtSOD1 transfected cells; G93A-SOD1, human G93A-SOD1 transfected cells. Endogenous mouse SOD1 (mSOD1) immunoreactivity was detected in all samples, whereas human SOD1 (hSOD1) was detected only in transfected samples. (e) Optical density analysis on human SOD1 accumulation. The histogram is obtained from dots optical densities of experiments performed in triplicate. *t*-Test analysis on wtSOD1 versus G93A-SOD1 transfected cells: *p < 0.05 versus wtSOD1 transfected C2C12 cells. ***p < 0.001 versus wtSOD1 transfected C2C12-D (differentiating) cells. (f) Flow cytofluorimetric analysis of total levels of GFP-tagged wt and mutant SOD1 expressed in NSC34, C2C12 and C2C12-D cells, normalized for the total number of GFP-positive cells to exclude variation as a result of different transfection efficiencies. *t*-Test analysis on wtSOD1 versus G93A-SOD1 transfected cells: ***p < 0.001 versus wtSOD1 C2C12 and C2C12-D transfected cells. (g) Filter retardation assay performed on cell lysates of NSC34 or C2C12 cells transfected with wt- or G93A-SOD1, in basal condition or after treatment with 10 μM of MG132 for 15 h. The histogram is obtained from dots optical densities of experiments performed in triplicate (NSC34 cells: **p < 0.01 wtSOD1 versus G93A-SOD1, ^###^*p* < 0.001 wtSOD1 + MG132 versus G93A-SOD1 + MG132, ^δδδ^*p* < 0.001 G93A-SOD1 versus G93A-SOD1 + MG132; C2C12 cells: ^##^*p* < 0.01 wtSOD1 + MG132 versus G93A-SOD1 + MG132, ^δδ^*p* < 0.01 G93A-SOD1 versus G93A-SOD1 + MG132; C2C12-D: ^###^*p* < 0.001 wtSOD1 + MG132 versus G93A-SOD1 + MG132, ^δδδ^*p* < 0.001 G93A-SOD1 versus G93A-SOD1 + MG132). (h) Flow cytofluorimetric analysis of MitoSOX-positive cells. NSC34, C2C12 and C2C12-D cells were transfected with GFP tagged SOD1, wt or G93A, and after labelled with MitoSOX reagents. The cytofluorimeter counts the number of red positive cells that were also GFP positive. GFP λ_ex_ = 488 nm, λ_em_ = 509 nm; MitoSOX Red λ_ex_ = 584 nm, λ_em_ = 607 nm. *t*-Test analysis on wtSOD1 versus G93A-SOD1 transfected cells: ^***^p < 0.0001 versus wtSOD1 NSC34 transfected cells.

We initially investigated whether mutant SOD1 forms intracellular aggregates in cultured muscle cells. Figure 1a shows that wtSOD1 is diffusely distributed both in motoneurons and in muscle C2C12 cells (either in undifferentiated/proliferating or differentiating conditions, C2C12 or C2C12-D respectively). As already reported, mutant G93A-SOD1 is excluded from the nuclei and accumulated into aggregates in motoneuronal NSC34 cells (Sau *et al.* 2007). On the contrary, in muscle C2C12 and C2C12-D cells the distribution of the mutant G93A-SOD1 was very similar to that observed with wtSOD1. Almost no mutant SOD1 aggregates were found in these conditions, indicating that intracellular processing of misfolded mutant G93A-SOD1 differs between neuronal and muscle cells. As fluorescence microscopy analysis indicated that the number of mutant SOD1-positive cells differs between NSC34 and C2C12 cells (data not shown), we wanted to quantify the transfection efficiency in the three cell types included in the study. This has been estimated by transfecting the soluble EGFP and counting fluorescence-positive cells in cytofluorimetric analysis (Fig. 1b). It clearly appears that proliferating C2C12 cells are transfected with an efficiency which is approximately 25% less that NSC34, whereas differentiating C2C12 cell transfection efficiency is about half of that measured in NSC34 cells. Keeping this in mind, we quantified the percentage of cells containing aggregates by a direct estimation of an identical large number of GFP-SOD1s transfected cells in fluorescence microscopy analysis. The data (Fig. 1c) demonstrated that about 30% of total transfected motoneuronal NSC34 cells contained intracellular mutant SOD1 aggregates, while only a negligible percentage of muscle C2C12 cells (either prior or during differentiation) were positive for intracellular mutant G93A-SOD1 aggregates. No aggregates have been observed in wtSOD1 expressing cells. Western blot analysis (Fig. 1d) revealed that in all cell type tested the levels of the monomeric mutant SOD1 is lower than that of the wtSOD1. The quantification of the monomeric mutant SOD1 levels in the three cell types considered is reported in Fig. 1e. Because of these dramatic differences in wt versus mutant SOD1 levels and aggregation rate found in NSC34 and C2C12 cells, these effects cannot be simply due to transfection efficiency (as previously estimated with the EGFP cytofluorimetric analysis, see Fig. 1b). Together these data suggest that the reduced levels of monomeric mutant SOD1 might be related to either a partial sequestration into insoluble oligomeric species (Fig. 1c) or to its increased cellular clearance or to both phenomena. In the case of motoneuronal NSC34 cells, we have already demonstrated that, intracellular accumulation of misfolded proteins may generate different multi-homomeric or heteromeric species, visible in fluorescence microscopy as inclusions (Sau *et al.* 2007; Crippa *et al.* 2010b).

To better clarify this point, we also performed cytofluorimetric experiments to measure the total levels (monomeric plus oligomeric species) of GFP-tagged SOD1s. The analysis considered only GFP-positive transfected cells in order to normalize for transfection efficiency. The results are reported in Fig. 1f, and show that, in NSC34 cells, in contrast to the data obtained for the monomeric SOD1 in western blot, the total amount of mutant SOD1 remained unchanged when compared with wtSOD1. On the contrary, the amount of total mutant SOD1 was dramatically reduced when compared with wtSOD1 in both C2C12 and C2C12-D cells. These data strongly support the notion that mutant SOD1 clearance is higher in C2C12 cells, than in NSC34 cells. To further support this observation, we quantify the total insoluble species of mutant SOD1 using the filter retardation assay. This experiment excludes that, in muscle C2C12 cells, mutant SOD1 might generate low-molecular weight insoluble species which are not in the aggregate form, because these species might have a limited size that does not allow their identification in fluorescence microscopy analysis. The data, reported in Fig. 1g, demonstrated that large amounts of insoluble mutant SOD1 accumulated in motoneuronal NSC34 cells already in basal conditions. Mutant SOD1 insoluble species massively accumulated when proteasome activity was blocked with the proteasome inhibitor MG132. This supports the notion that proteasome is involved in mutant misfolded SOD1 degradation. However, both in proliferating and differentiating muscle C2C12 cells, mutant SOD1 accumulation was comparable to the very low levels found for wtSOD1, in basal conditions. In muscle cells, mutant SOD1 insoluble species were observed only after MG132-mediated proteasome blockage. Thus, muscle C2C12 cells are able to correctly handle the fraction of misfolded mutant SOD1 that usually accumulate in immortalized motoneurons. The degradative system in muscle C2C12 cells not only prevents the formation of large misfolded protein inclusions, but also prevents the accumulation of species of smaller size (microaggregates). The reduction of monomeric mutant SOD1 (determined for C2C12 cells in Fig. 1d and e) must be due to an increased clearance of the misfolded protein that prevents its aggregation. Moreover, the fact that proteasome inhibition resulted in insoluble species accumulation proves that, also in muscle cells, the proteasomal degradative system is involved in the clearance of misfolded SOD1.

As NSC34 and C2C12 cells differ in their capacity to remove mutant SOD1, we wanted to determine whether this results in a selective alteration of the mitochondria, a well known target of mutant SOD1 neurotoxicity in ALS (Pasinelli and Brown 2006; Pedrini *et al.* 2010). To test this hypothesis, we analysed MitoSOX™ Red oxidation induced by superoxide. In fact, once in the mitochondria, MitoSOX exhibits red fluorescence selectively after its oxidation by superoxide, but not by other oxygen and/or nitrogen reactive species. Importantly, the MitoSOX oxidation is prevented by the SOD1 activity. The results are shown in Fig. 1h. It appears that, in NSC34 cells, a significant increase in MitoSOX oxidation is detectable in presence of mutant SOD1 compared with wtSOD1. No difference is observed in the MitoSOX oxidation in C2C12 cells expressing either wt or mutant SOD1, both in their proliferating or differentiating status. This clearly indicated that NSC34 cells expressing mutant SOD1 are more sensitive to a superoxide-induced oxidative stress than wtSOD1 cells (see also Sau *et al.* 2007; Crippa *et al.* 2010b). The removal of mutant SOD1 from C2C12 cells seems to protect against this toxic insult.

### Comparison of mutant SOD1 effects on proteasomal activity in motoneurons and muscle cells

**Fig. 2:**
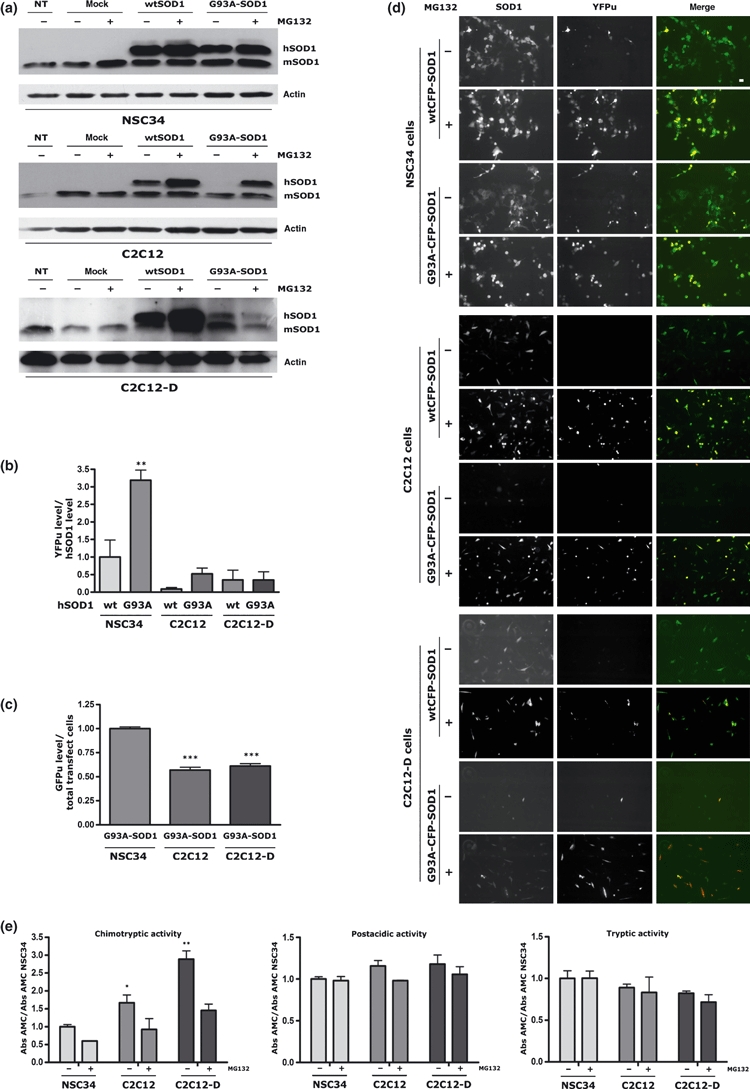
Involvement of the proteasome in mutant SOD1 clearance in motoneuronal NSC34 and muscle C2C12 cells. (a) Western blot assay performed on lysates of NSC34 or C2C12 cells (proliferating or differentiating, D), in basal condition or after treatment with 10 μM of MG132 for 15 h, to inhibit proteasome activity. Cells were transfected with wt- or G93A-SOD1. NT, untransfected cells; mock, pcDNA3 transfected cells; wtSOD1, human wtSOD1 transfected cells; G93A-SOD1, human G93A-SOD1 transfected cells. (b) Optical density analysis of western blot assay on NSC34 cells, proliferating or differentiating C2C12 cells (C2C12 and C2C12-D respectively) co-transfected with hSOD1s, wt or G93A, and YFPu plasmids (a proteasome activity reporter protein). The histogram is obtained from dots optical densities of experiments performed in triplicate, and normalized YFPu protein level with the corresponding monomeric hSOD1 protein. **p < 0.01 versus wtSOD1 transfected NSC34 cells. (c) Flow cytofluorimetric analysis on NSC34, C2C12 and C2C12-D cells co-transfected with mutant G93A-SOD1s, GFPu and DsRed-Monomer plasmids. GFPu fluorescence intensity was normalized with the number of transfected cells, evaluated as Red-positive cells. GFP λ_ex_ = 488 nm, λ_em_ = 509 nm; DsRed-Monomer λ_ex_ = 557 nm, λ_em_ = 592 nm. ***p < 0,001 versus G93A-SOD1 NSC34 expressing cells. (d) Fluorescent microscopy analysis on cells co-transfected with CFP-wtSOD1 or CFP-G93A-SOD1, and YFPu plasmids; cells were analysed in basal condition or after treatment with 10 μM of MG132 for 15 h, to inhibit proteasome activity. Upper insets = NSC34 cells; middle insets = proliferating C2C12 cells; lower insets = differentiating C2C12 (C2C12-D) cells. CFP λ_ex_ = 439 nm, λ_em_ = 476 nm; YFP λ_ex_ = 514 nm, λ_em_ = 527 nm. Images taken at 10×. Scale bar = 10 μm. (e) Analysis of proteasome activity in motoneuronal NSC34 and in muscle C2C12 cells, using specific AMC-conjugated peptides. Upper inset = chimotryptic activity; middle inset = post-acidic activity; lower inset = tryptic activity. Chimotryptic activity: *p < 0.05 NSC34 cells versus proliferating C2C12 cells; **p < 0.01 NSC34 cells versus differentiating C2C12 cells.

On this bases, we decided to further analyse the role of proteasome in the removal of mutant SOD1. Initially, we focus our attention on the levels of sodium dodecyl sulfate-soluble SOD1 (monomeric species detectable at the correct M.W. in western blot analysis. Fig. 2a). We confirmed that, in motoneuronal NSC34 cells (Fig. 2a, upper inset) the total amount of monomeric mutant SOD1 was slightly lower than that detected for wtSOD1. Conversely, in proliferating muscle C2C12 cells (Fig. 2a, middle inset), in absence of MG132 (with a fully functional proteasome system), the levels of mutant SOD1 were found to be greatly reduced when compared with those measured for wtSOD1. This difference was abolished when the clearance of wt or mutant SOD1 was blocked by proteasome inhibition. In fact, we found that proteasome inhibition with MG132 in NSC34 and proliferating C2C12 resulted in an increased level of wt or mutant SOD1s. This effect was particularly evident in proliferating C2C12 cells expressing mutant SOD1, suggesting that the very low mutant SOD1 levels found in basal condition, in these cells, are possibly caused by an higher protein clearance mediated by the proteasome (Fig. 2a).

Surprisingly, in differentiating muscle C2C12, proteasome inhibition resulted in a dramatic increase of the wtSOD1, whereas mutant SOD1 levels were not increased when proteasome was blocked (Fig. 2a, C2C12-D lower inset); this is apparently in contrast with the data showing an accumulation of insoluble mutant SOD1 after proteasome inhibition in the same type of cells (compare with Fig. 1g). It is conceivable that, in these conditions, an alternative pathway of degradation is highly active to remove the soluble monomeric mutant SOD1 fraction during proteasome blockage (see below).

As SOD1 clearance seems to involve the proteasome system, we then determined whether expression of mutant SOD1 results in alteration of the proteasome functions in immortalized motoneurons and in muscle cells. To this purpose, we utilized a proteasome activity reporter, the yellow fluorescent protein containing a constitutive degron signal (CL-1) (YFPu) (Bence *et al.* 2001; Rusmini *et al.* 2007, 2010). Alterations of the proteasome activity correlate with an accumulation of the YFPu in cells (Sau *et al.* 2007; Crippa *et al.* 2010b). The data obtained are shown in Fig. 2b. In this quantitative analysis, the YFPu levels (measured in western blot) were normalized by the corresponding intracellular levels of monomeric SOD1 (either wt or mutated) to reduce variations due to SOD1 clearance in NSC34, C2C12 and C2C12-D. In agreement with our previous reports *(*Sau *et al.* 2007; Crippa *et al.* 2010a; b), we found that YFPu accumulated in motoneuronal NSC34 cells expressing mutant SOD1. By contrast, mutant SOD1 expression in muscle C2C12 cells (either proliferating or differentiating) did not result in significant YFPu accumulation. This indicates that misfolded mutant SOD1 turnover alters proteasome functions only in immortalized motoneurons, but not in muscle C2C12 cells. To better evaluate the variation of proteasome functions in presence of mutant SOD1 in the three cell types considered, we also set up a cytofluorimetric analysis of proteasome activity. In this case, we used a different proteasome reporter with similar properties, the GFPu, and measured the fluorescence levels in cells expressing mutant SOD1, but normalized for the number of transfected cells; this allowed to avoid mis-interpretation caused by different transfection efficiency in the three cell types considered. The data showed in Fig. 2c, confirmed that the impact of mutant SOD1 on proteasome activity is very high in motoneuronal NSC34 cells, but low in muscle C2C12 cells. Overlapping results were obtained using fluorescence microscopy analysis (Fig. 2d). In fact, although high levels of YFPu were detectable in immortalized motoneurons expressing mutant SOD1, very few differentiating muscle cells resulted positive for YFPu. It might be possible that proteasome activity in muscle cells is higher than in motoneuronal cells, and this will help to quickly remove the misfolded mutant SOD1. Alternatively, other degradative systems might be activated in muscle cells in response to the presence of misfolded proteins. We thus tested the basal functions of the three different proteasome activities (chimotryptic, post-acidic and tryptic activities) in immortalized motoneurons in comparison to muscle C2C12 cells in the proliferating or differentiating status. The results illustrated in Fig. 2e, showed that the post-acidic and tryptic proteasome activities were comparable in all the three types of cultured cells considered. Interestingly, the chimotryptic activity was significantly higher in proliferating muscle C2C12 cells than in motoneuronal NSC34 cells; muscle C2C12 cells differentiation (C2C12-D) resulted in a further increase of the chimotryptic proteasome activity. As previously reported, only the chimotryptic activity is inhibited by the MG132, whereas the post-acidic and tryptic activities remained unchanged in presence of this proteasome inhibitor (Alexandrova *et al.* 2008).

### Comparison of the effects of mutant SOD1 on the autophagic system in motoneuron and muscle cells

**Fig. 3:**
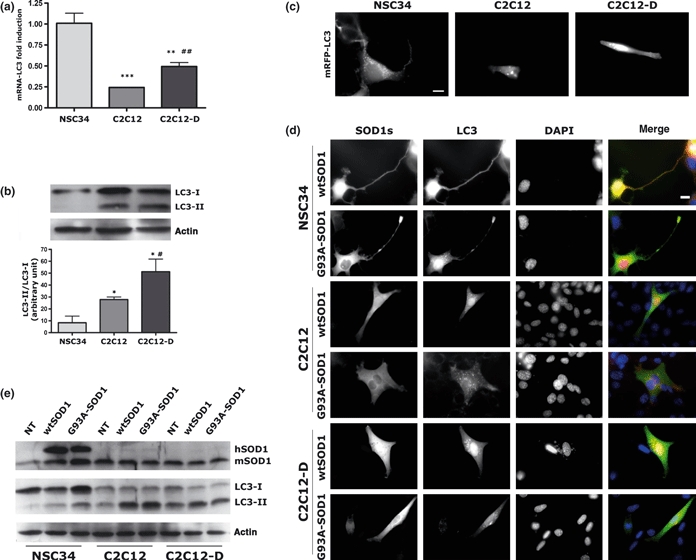
Autophagic pathway in motoneuronal NSC34 and muscle C2C12 cells. (a) Real-time PCR analysis of LC3- mRNA levels in basal condition in NSC34 cells and in C2C12 cells, proliferating or differentiating (D). ***p < 0.001 proliferating C2C12 cells versus NSC34 cells; **p < 0.01 differentiating C2C12 (C2C12-D) cells versus NSC34 cells; ^##^*p* < 0.01 differentiating (D) C2C12 cells versus proliferating C2C12 cells. (b) Western blot assay of LC3 proteins: lipidated (LC3-II) and unlipidated (LC3-I) LC3 in NSC34 and C2C12, proliferating and differentiating (D) cells. The histogram is obtained from optical densities of experiments performed in triplicate. To evaluate autophagy activation, we analysed the ratio of LC3-II and LC3-I. *p < 0.05 proliferating C2C12 cells and differentiating C2C12 (C2C12-D) cells versus NSC34 cells; ^#^*p* < 0.05 differentiating (D) C2C12 cells versus proliferating C2C12 cells. (c) High resolution fluorescent microscopy analysis of cells transfected with mRFP-LC3. mRFP λ_ex_ = 584 nm, λ_em_ = 607 nm. Images taken at 63×. (d) High resolution fluorescent microscopy analysis of NSC34 and C2C12 cells co-transfected with GFP-wtSOD1 or GFP-G93A-SOD1 and mRFP-LC3. Nuclei are stained with DAPI (blue). Upper insets = NSC34 cells; middle insets = proliferating C2C12 cells; lower insets = differentiating (D) C2C12 cells. GFP λ_ex_ = 488 nm, λ_em_ = 509 nm; mRFP λ_ex_ = 584 nm, λ_em_ = 607 nm; DAPI λ_ex_ = 358 nm, λ_em_ = 461 nm. Scale bar = 10 μm. (e) Western blot assay of LC3 protein in NSC34 and C2C12, proliferating and differentiating (D), cells. LC3-I is the unlipidated form, LC3-II is the lipidated form of LC3. NT, untransfected; wtSOD1, human wtSOD1 transfected cells; G93A-SOD1, human G93A-SOD1 transfected cells.

The proteasome system is one of the major intracellular degradative pathway and acts in synergy with the autophagic system. We thus evaluated the involvement of macroautophagy by analysing the expression levels of LC3, a well known marker of this degradative pathway. LC3 mRNA expression levels were tested in motoneuronal NSC34, and in muscle C2C12 cells (either in the proliferating or differentiating status). The results are shown in Fig. 3a, and clearly indicate that motoneuronal NSC34 cells expressed about 3 times more LC3-mRNA than muscle C2C12 cells grown in basal condition. Muscle C2C12 cells differentiation (C2C12-D) resulted in a two-fold increase of the LC3-mRNA expression levels respect to the undifferentiated (proliferating) cells. Therefore, while motoneuronal NSC34 cells possess a lower proteasome activity than muscle C2C12 cells, motoneuronal NSC34 cells are characterized by a much higher potential induction of the autophagic process, indicated by the increased levels of LC3-mRNA. However, although LC3 over-induction may act as permissive factor in autophagy, LC3 protein processing to its lipidated form LC3-II is absolutely required to achieve autophagy activation. In fact, to permit the complete flow of the autophagosomes from their formation to the fusion with the lysosomes, the autophagosomes must be decorated by the lipidated LC3-II anchored to the membrane via the phosphatidylethanolamine (PE) tail (Garcia-Arencibia *et al.* 2010). Therefore, constitutive activation of autophagy in the three cell types was studied by analysing the formation of the lipidated LC3-II form in western blot analysis, and then by determining the LC3-II/LC3-I ratio which is indicative of the levels of autophagy in the cells (Rubinsztein *et al.* 2009; Mizushima *et al.* 2010). The data are shown in Fig. 3b. Differentiating muscle C2C12 cells (C2C12-D) were characterized by a very high activation of the autophagic pathway, because the ratio between LC3-II and LC3-I was largely in favour of the autophagosome-anchored lipidated LC3-II form. This ratio appeared to be much higher than that observed in proliferating muscle C2C12 cells. The constitutive activation of autophagy in motoneuronal NSC34 cells was negligible, if compared with the one in the muscle cell types. We then validated these observations by analysing the intracellular distribution of exogenous LC3 (mRFP-LC3) in fluorescence microscopy. In basal conditions, LC3 is normally diffused into the cytoplasm, whereas during autophagy activation it acquires a punctate distribution caused by its lipidation and association to the autophagosomes. Figure 3c shows the presence of largely diffused LC3 distribution in motoneuronal NSC34 cells, with the presence of some autophagosomes, indicative of basal levels of autophagic activation. Instead, in muscle C2C12 cells, LC3 showed a more punctate distribution, confirming that in muscle cells the basal autophagic activity is higher than in immortalized motoneurons.

After a characterization of autophagy in basal conditions in the three cell types, we analysed the effects of wt or mutant SOD1 on this degradative pathway. The results are shown in Fig. 3d. It clearly appears that the expression of the mutant SOD1 in motoneuronal NSC34 cells correlated with a LC3 distribution similar to that found for wtSOD1, mainly in a diffused form, with a slight increase of the LC3 in the punctate distribution; this suggests the existence of a limited autophagy activation in the presence of misfolded SOD1. In muscle C2C12 cells, both in the proliferating and differentiating status, constitutive autophagic activation was confirmed to be already present in wtSOD1 expressing cells (in agreement with the data of Fig. 3c). However, mutant SOD1 over-expression correlated with a dramatic increase of the punctate LC3 status indicating a potent activation of the autophagic process in the presence of the misfolded mutant SOD1. Moreover, in agreement with our western blot analysis, the total levels of mutant SOD1 in muscle C2C12 cells were found greatly reduced in comparison to those found in motoneuronal NSC34 cells. These data were then validated with western blot analyses reported in Fig. 3e, where we observed an increase of LC3-II in cells expressing hSOD1s.

**Fig. 4:**
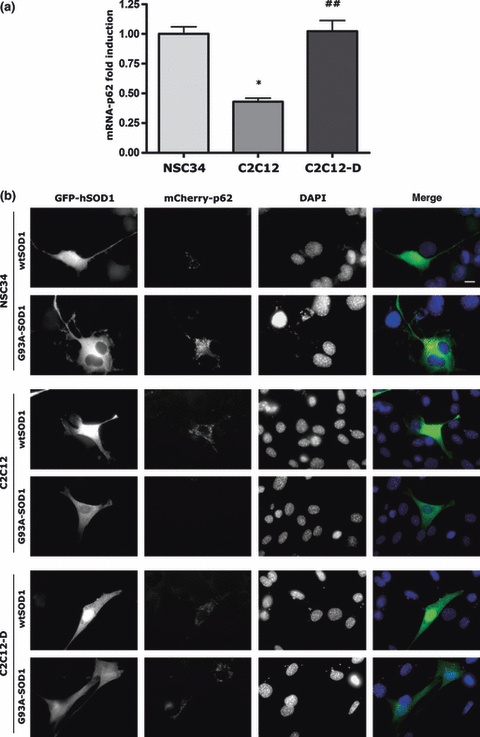
p62 expression and localization. (a) Real-time PCR analysis of mRNA-p62 levels in basal condition in NSC34 cells and in C2C12 cells, proliferating or differentiating (D). *p < 0.05 proliferating C2C12 cells versus NSC34 cells; ^#^*p* < 0.01 differentiating (D) C2C12 cells versus C2C12 cells. (b) High resolution fluorescent microscopy analysis of NSC34 and C2C12 cells co-transfected with GFP-wtSOD1 or GFP-G93A-SOD1 and mCherry-p62. Nuclei are stained with DAPI (blue). Upper insets = NSC34 cells; middle insets = proliferating C2C12 cells; lower insets = differentiating (D) C2C12 cells. GFP λ_ex_ = 488 nm, λ_em_ = 509 nm; mCherry λ_ex_ = 587 nm, λ_em_ = 610 nm; DAPI λ_ex_ = 358 nm, λ_em_ = 461 nm. Images taken at 63×. Scale bar = 10 μm.

Because of the mutant SOD1 induced autophagy present in muscle C2C12 cells, but limited in motoneuronal NSC34 cells, we decided to further investigate the activation of the autophagic system using an alternative autophagic marker, the p62. This protein is responsible for the recognition of ubiquitinated misfolded substrates, and for their insertion into the nascent autophagosome. Moreover, p62, as well as LC3, is transcriptionally up-regulated during autophagy (Nakano *et al.* 2004; Nakaso *et al.* 2004; He and Klionsky 2009). In addition, it is known that both p62 mRNA and protein levels increased after cells damages, inhibition of proteasome activity, presence of misfolded proteins (Bjorkoy *et al.* 2006). Thus, p62 is considered a marker for the potential of a given cell to respond to these aberrant stimuli. Interestingly, when the autophagic flux is blocked, the p62 protein levels increased as a result of the blockage of its degradation (Klionsky *et al.* 2008). On these bases, we initially compared the expression levels of p62-mRNA in the three cell types considered, to evaluate their potential response to aberrant stimuli. The results obtained (Fig. 4a) showed that, in basal conditions, proliferating muscle C2C12 cells were characterized by p62-mRNA levels significantly lower than motoneuronal NSC34 cells. However, after being induced to differentiate, C2C12 cells were found to express p62-mRNA levels comparable to that found in motoneuronal NSC34 cells. The expression of wt or mutant SOD1 either in motoneuronal NSC34 cells or in C2C12 cells (proliferating or differentiating) did not significantly modify the expression levels either of the endogenous p62 or LC3 (data not shown). We thus analysed the possible accumulation and the intracellular distribution of exogenous p62 (mCherry-p62) in immortalized motoneurons or muscle cells expressing wt or mutant SOD1. The results (Fig. 4b) showed that mutant SOD1 expression in motoneuronal NSC34 cells correlated with a dramatic accumulation of mCherry-p62, while these levels were much lower in wtSOD1 expressing motoneuronal NSC34 cells. On the contrary, the mCherry-p62 levels remained very low in muscle C2C12 cells expressing either wt or mutant SOD1, both in proliferating and in differentiating status. These data strongly agree with those obtained using LC3 and suggest that mutant SOD1 expression results in autophagic flux blockage only in immortalized motoneuronal NSC34 cells (Klionsky *et al.* 2008).

## Discussion

In the present study, we analysed whether fALS-related mutant SOD1 has different biochemical behaviour in motoneuronal and muscle cells, as well as the relative contribution of the two major intracellular degradative pathways involved in mutant SOD1 clearance. We found that muscle cells more efficiently remove misfolded mutant SOD1 than motoneuronal cells. In fact, mutant SOD1 aggregates can be easily observed in motoneuronal cells, both in fluorescence microscopy and in filter retardation assay, whereas only negligible amounts of insoluble mutant SOD1 are detectable either in proliferating or differentiating muscle cells. These data are paralleled by observations showing that the relative degradation rate of mutant SOD1, compared with wtSOD1, is lower in motoneuronal cells than in muscle cells (both in proliferating and differentiating status); instead, in muscle cells, mutant SOD1 clearance is very high. Interestingly, proteasome inhibition resulted in the accumulation of misfolded mutant SOD1 in all cell types, suggesting that proteasome plays an important role in the removal of the mutant protein both in motoneurons and in muscle cells. However, mutant SOD1 expression leads to an impairment of the proteasome activity only in motoneuronal cells, and not in muscle cells. This is demonstrated by the selective accumulation of the proteasome reporter, YFPu, in motoneuronal NSC34 cells, but not in muscle C2C12 cells (both in proliferating and differentiating status). Interestingly, we found that muscle C2C12 cells are characterized by a much higher chimotryptic proteasome activity than motoneuronal NSC34 cells, whereas the other two proteolytic activities of the proteasome were comparable. Therefore, the increased mutant misfolded SOD1 turnover in muscle cells might be, at least in part, due to the much higher chimotryptic proteasome activity, typical of these cells. Notably, the chimotryptic activity greatly increased after muscle cells differentiation, when the levels of mutant SOD1 appeared to be greatly reduced, in comparison to proliferating (undifferentiated) muscle cells. Moreover, MG132 inhibitory activity is directed only against the chimotryptic activity of the proteasome (Alexandrova *et al.* 2008), with no effect of the two other proteolytic activity associated to this pathway, and mutant SOD1 accumulates after MG132 treatment, supporting the notion that the chimotryptic proteasome activity is the one responsible for the clearance of the mutant SOD1 through this pathway.

Our previous data (Crippa *et al.* 2010b) demonstrated that mutant SOD1 can be removed, not only by the proteasome, but also by alternative pathways of degradation, such as autophagy. Surprisingly, here we found that motoneuronal NSC34 cells are characterized by a very high mRNA expression of the autophagic mediator LC3. In fact, LC3-mRNA levels were found to be three times higher in motoneuronal NSC34 cells than in proliferating muscle C2C12 cells. LC3-mRNA slightly increased with muscle cell differentiation, because it was twofold higher in motoneuronal NSC34 cells than in differentiating muscle C2C12 cells. Despite this, LC3 activation (conversion to the lipidated LC3-II forms and to a punctate distribution) was much higher in muscle C2C12 cells (both in proliferating and differentiating conditions) than in motoneuronal NSC34 cells. This indicates that autophagic system is activated at high levels in muscle cells, and is poorly activated in motoneuronal NSC34 cells. Therefore, the higher LC3-mRNA expression, combined with the low activation of LC3 observed in motoneuronal NSC34 cells, apparently indicates that these cells have the potential to activate the autophagic system, but the efficiency of the activation is much lower than in muscle C2C12 cells. The high LC3-mRNA expression in motoneuronal NSC34 cells might be interpreted as the response of the cells to satisfy a reduced potential of these cells to activate autophagy (Rubinsztein *et al.* 2009; Arndt *et al.* 2010; Garcia-Arencibia *et al.* 2010; Mizushima *et al.* 2010). Notably, mutant SOD1 expression correlated with a very high activation of LC3, in all cell types, but particularly in muscle C2C12 and C2C12-D cells in which almost all the LC3 was found in the lipidated form, the LC3-II, tightly associated to the newly formed autophagosomes.

The increased LC3 expression in motoneuronal NSC34 cells might be a result of a proteasome engulfment, which requires the activation of the alternative autophagic pathway, triggered by the accumulating misfolded proteins. By contrast, in muscle C2C12 cells, which are characterized by higher chimotryptic proteasome activity, we found that, the proteasome activity was not impaired by the mutant SOD1 (no accumulation of YFPu), thus the LC3 levels remained lower. It remains to be determined whether the autophagic flux is functional in the cell models considered.

The involvement of the autophagic pathway in the removal of mutant SOD1 from muscle cells has been then further confirmed by the analysis of p62 distribution in motoneuronal NSC34 and muscle C2C12 cells. In fact, in immortalized motoneuronal NSC34 cells, mutant SOD1 expression induced a dramatic accumulation of exogenous Cherry-labelled p62, whereas no effects were observed in muscle C2C12 cells (both in proliferating and in differentiating status) expressing mutant SOD1. This observation indicates that mutant SOD1 expression correlates with an autophagic flux blockage in immortalized motoneuronal NSC34 cells.

Therefore, muscle cells are apparently more protected than motoneuronal cells against accumulation of misfolded mutant SOD1 in fALS. However, Dobrowolny *et al.* (2008b) have recently undoubtely demonstrated in mice models of ALS that muscle is a primary target of mutant SOD1 toxicity (Dobrowolny *et al.* 2008b). In fact, the muscle-restricted expression of the mutant SOD1 (MLC/SOD1G93A) was sufficient to induce severe muscle atrophy, with a significant reduction in muscle strength, sarcomere disorganization, as well as mitochondrial alterations and disorganization of the sarcotubular system (Dobrowolny *et al.* 2008b). More recently, by producing a second transgenic MLC/SOD1G93A ALS model, Wong and Martin (2010) confirmed the observation of Dobrowolny and colleagues just described. In addition, they clearly demonstrated that the MLC/SOD1G93A in transgenic mice also correlated with the loss of spinal cord motoneurons that are devoid of mutant SOD1 (Wong and Martin 2010). Therefore, in ALS, motoneuron loss might also derive from the dysfunction occurring in the muscular cells, which are also target of mutant SOD1 toxicity. These data might appear in contrast with our observations showing that muscle cells respond to the presence of insoluble mutant SOD1 much better than motoneurons. A second possible explanation arises from very recent experimental data showing that the activity of the proteasome system in the skeletal muscle differs in individual muscle groups and more importantly decreases with aging (Strucksberg *et al.* 2010). Moreover, a 50% reduction in specific catalytic activity of the 20S proteasome was reported in aged rats, and a significant reduction in the proteasomal degradation of oxidized calmodulin was observed in aged muscle (Ferrington *et al.* 2005; Husom *et al.* 2005). The reduced proteasome activity seems to be due to alterations in the composition of the 20S proteasome complex with a 50% reduction of the activating complexes, PA28 and PA700 (Husom *et al.* 2005), suggesting that significant age-related changes in proteasome structure, function, and oxidation state that could inhibit removal of oxidized proteins (Ferrington *et al.* 2005; Husom *et al.* 2005). Unfortunately, these aspects cannot be evaluated in cultured muscle C2C12 cells. Finally, an alternative explanation includes the possible differences in mutant SOD1 aggregation in mitochondria of motoneurons or muscle cells. In fact, it has been recently shown that removal of aggregates in the cytosol of neuronal cells is not sufficient to rescue cells from damage induced by mutant SOD1 (Ferri *et al.* 2010), whereas the removal of mutant SOD1 mitochondrial aggregates has positive effects on cell survival (Ferri *et al.* 2010). Future studies should be devoted to analyse possible modification in mitochondria of motoneuron versus muscle cells. It could also be possible that insoluble/aggregated SOD1 is not the primary cause of toxicity. It has been proposed that other mechanisms (formation of aberrant free radical species, mitochondrial dysfunction, nuclear deprotection, etc.) (Bendotti and Carri 2004; Cozzolino *et al.* 2008, 2009) may play a relevant role in inducing cell dysfunctions both in motoneurons and muscle cells, independently from the presence of aggregates. In the transgenic ALS mice expressing mutant SOD1 in muscle cells, it has been demonstrated the activation of signals correlated with oxidative stress (Dobrowolny *et al.* 2008b): for example, activation of FoxO and NFkB, that are both capable to induce the expression of several atrophy-related genes. In the same animals, there was an accumulation of reactive oxygen species that serves as signalling to initiate autophagy. In fact, the muscle of these mice revealed the T-tubule as the potential donor of membrane that forms sequestering autophagocytic vesicles (Dobrowolny *et al.* 2008b).

All together these data indicate that muscle cells are more resistant to the accumulation of misfolded species of the mutant SOD1, but this might not be related to the cell toxicity exerted by this mutant protein.

## Abbreviations used

ALS: amyotrophic lateral sclerosis
AMC: aminomethylcoumarin
CFP: cyan fluorescent protein
fALS: familial ALS
GFP: green fluorescent protein
PBS: phosphate-buffered saline
SOD1: superoxide dismutase 1

## References

[b1] Alexandrova A, Petrov L, Georgieva A, Kirkova M, Kukan M (2008). Effects of proteasome inhibitor, MG132, on proteasome activity and oxidative status of rat liver. Cell Biochem. Funct..

[b2] Allen S, Heath PR, Kirby J, Wharton SB, Cookson MR, Menzies FM, Banks RE, Shaw PJ (2003). Analysis of the cytosolic proteome in a cell culture model of familial amyotrophic lateral sclerosis reveals alterations to the proteasome, antioxidant defenses, and nitric oxide synthetic pathways. J. Biol. Chem..

[b3] Arndt V, Dick N, Tawo R (2010). Chaperone-assisted selective autophagy is essential for muscle maintenance. Curr. Biol..

[b4] Bampton ET, Goemans CG, Niranjan D, Mizushima N, Tolkovsky AM (2005). The dynamics of autophagy visualized in live cells: from autophagosome formation to fusion with endo/lysosomes. Autophagy.

[b5] Batandier C, Fontaine E, Keriel C, Leverve XM (2002). Determination of mitochondrial reactive oxygen species: methodological aspects. J. Cell Mol. Med..

[b6] Bence NF, Sampat RM, Kopito RR (2001). Impairment of the ubiquitin-proteasome system by protein aggregation. Science.

[b7] Bendotti C, Carri MT (2004). Lessons from models of SOD1-linked familial ALS. Trends Mol. Med..

[b8] Bjorkoy G, Lamark T, Johansen T (2006). p62/SQSTM1: a missing link between protein aggregates and the autophagy machinery. Autophagy.

[b9] Cashman NR, Durham HD, Blusztajn JK, Oda K, Tabira T, Shaw IT, Dahrouge S, Antel JP (1992). Neuroblastoma x spinal cord (NSC) hybrid cell lines resemble developing motor neurons. Dev. Dyn..

[b10] Chomczynski P, Sacchi N (1987). Single step method of RNA isolation by acid guanidinium thyocyanate-phenol chloroform extraction. Anal. Biochem..

[b11] Cozzolino M, Ferri A, Carri MT (2008). Amyotrophic lateral sclerosis: from current developments in the laboratory to clinical implications. Antioxid. Redox Signal..

[b12] Cozzolino M, Pesaresi MG, Amori I, Crosio C, Ferri A, Nencini M, Carri MT (2009). Oligomerization of mutant SOD1 in mitochondria of motoneuronal cells drives mitochondrial damage and cell toxicity. Antioxid. Redox Signal..

[b13] Crippa V, Carra S, Rusmini P, Sau D, Bolzoni E, Bendotti C, De Biasi S, Poletti A (2010a). A role of small heat shock protein B8 (HspB8) in the autophagic removal of misfolded proteins responsible for neurodegenerative diseases. Autophagy.

[b14] Crippa V, Sau D, Rusmini P (2010b). The small heat shock protein B8 (HspB8) promotes autophagic removal of misfolded proteins involved in amyotrophic lateral sclerosis (ALS). Hum. Mol. Genet..

[b15] Dobrowolny G, Giacinti C, Pelosi L, Nicoletti C, Winn N, Barberi L, Molinaro M, Rosenthal N, Musaro A (2005). Muscle expression of a local Igf-1 isoform protects motor neurons in an ALS mouse model. J. Cell Biol..

[b16] Dobrowolny G, Aucello M, Molinaro M, Musaro A (2008a). Local expression of mIgf-1 modulates ubiquitin, caspase and CDK5 expression in skeletal muscle of an ALS mouse model. Neurol. Res..

[b17] Dobrowolny G, Aucello M, Rizzuto E (2008b). Skeletal muscle is a primary target of SOD1G93A-mediated toxicity. Cell Metab..

[b18] Dobson CM (2003). Protein folding and misfolding. Nature.

[b19] Ferri A, Fiorenzo P, Nencini M, Cozzolino M, Pesaresi MG, Valle C, Sepe S, Moreno S, Carri MT (2010). Glutaredoxin 2 prevents aggregation of mutant SOD1 in mitochondria and abolishes its toxicity. Hum. Mol. Genet..

[b20] Ferrington DA, Husom AD, Thompson LV (2005). Altered proteasome structure, function, and oxidation in aged muscle. FASEB J..

[b21] Fischer LR, Culver DG, Tennant P, Davis AA, Wang M, Castellano-Sanchez A, Khan J, Polak MA, Glass JD (2004). Amyotrophic lateral sclerosis is a distal axonopathy: evidence in mice and man. Exp. Neurol..

[b22] Frey D, Schneider C, Xu L, Borg J, Spooren W, Caroni P (2000). Early and selective loss of neuromuscular synapse subtypes with low sprouting competence in motoneuron diseases. J. Neurosci..

[b23] Garcia-Arencibia M, Hochfeld WE, Toh PP, Rubinsztein DC (2010). Autophagy, a guardian against neurodegeneration. Semin. Cell Dev. Biol.

[b24] He C, Klionsky DJ (2009). Regulation mechanisms and signaling pathways of autophagy. Annu. Rev. Genet..

[b25] Husom AD, Ferrington DA, Thompson LV (2005). Age-related differences in the adaptive potential of type I skeletal muscle fibers. Exp. Gerontol..

[b26] Kaspar BK, Llado J, Sherkat N, Rothstein JD, Gage FH (2003). Retrograde viral delivery of IGF-1 prolongs survival in a mouse ALS model. Science.

[b27] Klionsky DJ, Abeliovich H, Agostinis P (2008). Guidelines for the use and interpretation of assays for monitoring autophagy in higher eukaryotes. Autophagy.

[b28] Mizushima N, Yoshimori T, Levine B (2010). Methods in mammalian autophagy research. Cell.

[b29] Musaro A, McCullagh K, Paul A, Houghton L, Dobrowolny G, Molinaro M, Barton ER, Sweeney HL, Rosenthal N (2001). Localized Igf-1 transgene expression sustains hypertrophy and regeneration in senescent skeletal muscle. Nat. Genet..

[b30] Nakano T, Nakaso K, Nakashima K, Ohama E (2004). Expression of ubiquitin-binding protein p62 in ubiquitin-immunoreactive intraneuronal inclusions in amyotrophic lateral sclerosis with dementia: analysis of five autopsy cases with broad clinicopathological spectrum. Acta Neuropathol..

[b31] Nakaso K, Yoshimoto Y, Nakano T (2004). Transcriptional activation of p62/A170/ZIP during the formation of the aggregates: possible mechanisms and the role in Lewy body formation in Parkinson's disease. Brain Res..

[b32] Palazzolo I, Stack C, Kong L (2009). Overexpression of IGF-1 in muscle attenuates disease in a mouse model of spinal and bulbar muscular atrophy. Neuron.

[b33] Pasinelli P, Brown RH (2006). Molecular biology of amyotrophic lateral sclerosis: insights from genetics. Nat. Rev. Neurosci..

[b34] Pedrini S, Sau D, Guareschi S, Bogush M, Brown RH, Naniche N, Kia A, Trotti D, Pasinelli P (2010). ALS-linked mutant SOD1 damages mitochondria by promoting conformational changes in Bcl-2. Hum. Mol. Genet..

[b35] Rubinsztein DC, Cuervo AM, Ravikumar B, Sarkar S, Korolchuk V, Kaushik S, Klionsky DJ (2009). In search of an “autophagomometer”. Autophagy.

[b36] Rusmini P, Sau D, Crippa V, Palazzolo I, Simonini F, Onesto E, Martini L, Poletti A (2007). Aggregation and proteasome: the case of elongated polyglutamine aggregation in spinal and bulbar muscular atrophy. Neurobiol. Aging.

[b37] Rusmini P, Bolzoni E, Crippa V, Onesto E, Sau D, Galbiati M, Piccolella M, Poletti A (2010). Proteasomal and autophagic degradative activities in spinal and bulbar muscular atrophy. Neurobiol. Dis..

[b38] Sau D, De Biasi S, Vitellaro-Zuccarello L (2007). Mutation of SOD1 in ALS: a gain of a loss of function. Hum. Mol. Genet..

[b39] Seetharaman SV, Prudencio M, Karch C, Holloway SP, Borchelt DR, Hart PJ (2009). Immature copper–zinc superoxide dismutase and familial amyotrophic lateral sclerosis. Exp. Biol. Med. (Maywood).

[b40] Simeoni S, Mancini MA, Stenoien DL, Marcelli M, Weigel NL, Zanisi M, Martini L, Poletti A (2000). Motoneuronal cell death is not correlated with aggregate formation of androgen receptors containing an elongated polyglutamine tract. Hum. Mol. Genet..

[b41] Strucksberg KH, Tangavelou K, Schroder R, Clemen CS (2010). Proteasomal activity in skeletal muscle: a matter of assay design, muscle type, and age. Anal. Biochem..

[b42] Tortarolo M, Crossthwaite AJ, Conforti L, Spencer JP, Williams RJ, Bendotti C, Rattray M (2004). Expression of SOD1 G93A or wild-type SOD1 in primary cultures of astrocytes down-regulates the glutamate transporter GLT-1: lack of involvement of oxidative stress. J. Neurochem..

[b43] Wang L, Sharma K, Deng HX, Siddique T, Grisotti G, Liu E, Roos RP (2008). Restricted expression of mutant SOD1 in spinal motor neurons and interneurons induces motor neuron pathology. Neurobiol. Dis..

[b44] Wong M, Martin LJ (2010). Skeletal muscle-restricted expression of human SOD1 causes motor neuron degeneration in transgenic mice. Hum. Mol. Genet..

